# Structure Identification of Euphorbia Factor L3 and Its Induction of Apoptosis through the Mitochondrial Pathway

**DOI:** 10.3390/molecules16043222

**Published:** 2011-04-15

**Authors:** Jian-Ye Zhang, Yong-Ju Liang, Hu-Biao Chen, Li-Sheng Zheng, Yan-Jun Mi, Fang Wang, Xiao-Qin Zhao, Xiao-Kun Wang, Hui Zhang, Li-Wu Fu

**Affiliations:** 1 Department of Pharmaceutical Sciences, School of Basic Science, Guangzhou Medical College, Guangzhou 510182, China; 2 State Key Laboratory of Oncology in South China, Cancer Center, Sun Yat-Sen University, Guangzhou 510060, China; 3 Guangzhou Institute of Respiratory Diseases, State Key Laboratory of Respiratory Diseases, The First Affiliated Hospital, Guangzhou Medical College, Guangzhou 510120, China; 4 School of Chinese Medicine, Hong Kong Baptist University, Kowloon Tong, Hong Kong, China; Email: hbchen@hkbu.edu.hk

**Keywords:** euphorbia factor L3, caper euphorbia seed, apoptosis, mitochondrial pathway

## Abstract

In this article, we have focused on the structure identification of Euphorbia factor L3 belonging to the lathyrane diterpenoids isolated from Caper Euphorbia Seed. Its anticancer activity *in vitro* against lung cancer A549 cells was also investigated and the IC_50_ values were 34.04 ± 3.99 μM. Furthermore, Euphorbia factor L3 could induce apoptosis in A549 cells via the mitochondrial pathway including loss of mitochondrial potential and release of cytochrome *c*.

## 1. Introduction

Containing more than 2,000 species, the genus *Euphorbia* is the largest one in the spurge family. Some species of the genus *Euphorbia* have been used as medicinal plants for the treatment of cancer, migraine, gonorrhea, intestinal parasites and skin diseases. Furthermore, in Traditional Chinese Medicine, Caper Euphorbia Seed (seeds of *Euphorbia lathyris* L.) has traditionally been applied to treat cancer [1,2]. Euphorbia factors L1-L11, belonging to the lathyrane diterpenoids, have been isolated from Caper Euphorbia Seed. Lathyrane diterpenoids have been reported to show cytotoxicity to cancer cells and ability of reversing multidrug resistance (MDR) [1,3,4]. 

Previously, we reported the isolation, identification, anticancer activity of Euphorbia factor L1 from Caper Euphorbia Seed [2]. As part of our ongoing work, in this article the purification and structure identification of Euphorbia factor L3 (EFL3) were reported. Additionally, the anticancer activity against lung cancer cell line A549 was investigated *in vitro*. For the first time, EFL3 was found to show potent cytotoxicity and induce apoptosis via the mitochondrial pathway in A549 cells with involvement of loss of mitochondrial potential and release of cytochrome *c*.

## 2. Results and Discussion

**Figure 1:**
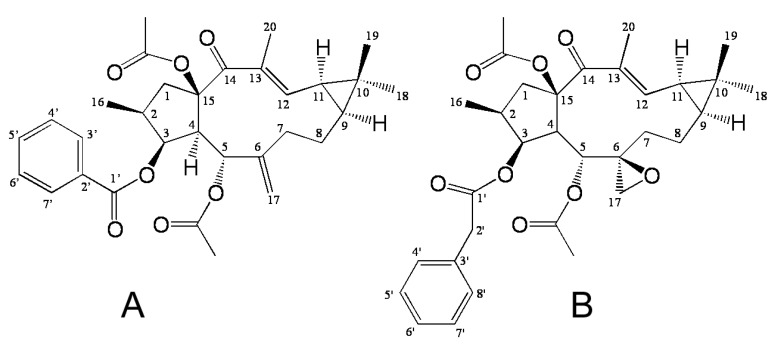
The chemical structure of Euphorbia factor L3 (EFL3, 1A) and Euphorbia factor L1 (EFL1, 1B).

EFL3 (Figure 1A) was obtained as colorless crystal from dichloromethane-petroleum ether. The molecular weight was determined by EIMS showing a peak at *m/z* 522. Its IR spectrum exhibited absorptions at 2925, 1739, 1714, 1650, 1622, 1452, 1370, 1277, 1222, 1109 and 712 cm^−1^. The ^1^H- NMR (Table 1) displayed five benzene protons with the chemical shifts between 7.26 and 8.04, which implied presence of one benzene ring. In the ^13^C-NMR (Table 1), 29 carbon signals were observed. This compound might contain 31 carbons based on collective consideration of the benzene ring. The ^13^C-NMR showed four carbonyls of 196.72, 170.13, 169.65 and 166.13, respectively. It was identified as Euphorbia factor L3 (C_31_H_38_O_7_, EFL3) after comparison with the NMR data of reference data [3,5]. 

**Table 1:** ^1^H and ^13^C NMR spectral data (400 and 100 MHz, δ in ppm, multiplicities, J in Hz).

Position		^13^C	^1^H
1		48.56	3.52（dd，1H，8，14）
			1.66（dd，1H，11，14）
2		37.93	2.36（m，1H）
3		80.87	5.82（t，1H，3.2）
4		52.24	2.90（dd，1H，3.2，10）
5		65.48	6.21（d，1H，10）
6		144.61	
7		34.94	2.18（m，1H）
			2.05（m，1H）
8		21.67	1.94（m，1H）
			1.72（m，1H）
9		35.39	1.15（m，1H）
10		25.26	
11		28.99	1.40（dd，1H，8.5，11.5）
12		146.43	6.54（dd，1H，1，11）
13		134.24	
14		196.72	
15		92.51	
16		14.18	0.94（d，3H，6.5）
17		115.40	5.01（s，1H）
			4.77（s，1H）
18		28.55	1.17（s，3H）
19		16.82	1.16（s，3H）
20		12.45	1.72（s，3H）
5-OAc	CH_3_	20.92	1.26（s，3H）
	CO	169.65	
15-OAc	CH_3_	21.95	2.21（s，3H）
	CO	170.13	
3-OBz	1’	166.13	
	2’	130.15	
	5’	133.08	7.56（m，1H）
	3’，7’	129.63	8.04（m，2H）
	4’，6’	128.30	7.45（m，2H）

**Figure 2:**
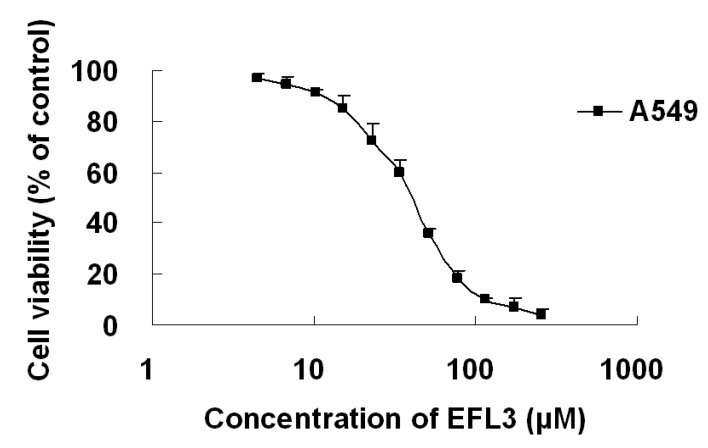
The IC_50_ curve against A549 cells. A549 cells were treated with indicated concentrations of EFL3 for 72 h. Each point represents the means ± standard deviations (SDs) of three determinations. Each experiment was performed in three replicate wells.

The cytotoxicity of EFL3 was determined by an MTT assay to evaluate its antitumor activity. The results showed that EFL3 inhibited cell proliferation in a concentration-dependent manner in A549 cells after 72 h treatment (Figure 2). The IC_50_ of EFL3 for A549 cells was 34.04 ± 3.99 μM. The data suggested that EFL3 exhibited potent cytotoxicity towards these cells. Also, cytotoxicities (IC_50_ values) against MCF-7 and LoVo cells were 45.28 ± 2.56 and 41.67 ± 3.02 μM, respectively. The statistical analysis showed that the IC_50_ values against A549 were less than those against MCF-7 and LoVo cells (*P* < 0.05). Thus, A549 cells were selected in this study. Additionally, cytotoxicity of Euphorbia factor L1 (EFL1, Figure 1B) against A549 cells was investigated to make comparison and the IC_50_ values were 51.34 ± 3.28 μM. The results showed that EFL3 showed more potent cytotoxicity to A549 cells than EFL1 (*P* < 0.01). We tried to make a structure activity analysis based on these results. The chemical structure differences between EFL1 and EFL3 are the 3-phenylacetoxy and 6(17)-epoxide moieties for EFL1 and 3-benzoyloxy and 6(17)-ene for EFL3. It is quite possible that three-ring strain of 6(17)-epoxide has a disadvantageous role as far as the cytotoxicity is concerned.

Many anticancer drugs, such as anthracyclines, may induce apoptosis and kill susceptible tumor cells [6]. To clarify whether EFL3 induced cell apoptosis of A549 cells, the Annexin-V and PI double staining were performed. 

**Figure 3:**
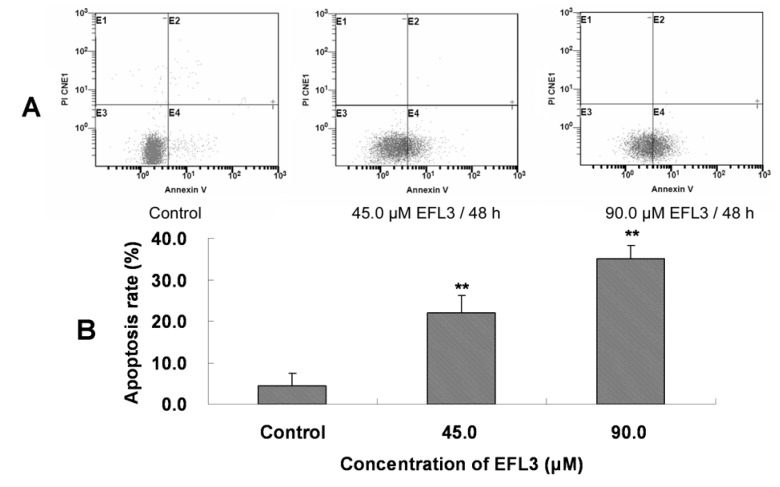
EFL3-mediated apoptosis in A549 cells was detected by Annexin V-FITC/PI double staining and flow cytometer. **(A)** E4 quadrant represented cells stained mainly by Annexin-V (early apoptotic cells) and the E2 quadrant represented cells stained by both PI and Annexin-V (late apoptotic). The E1 quadrant represented cells stained mainly by PI and viable cells negative for both Annexin-V and PI appeared in the E3 quadrant. **(B)** The total apoptosis rate was exhibited in the bar graph. ^*^P < 0.05 and ^**^P < 0.01 *vs.* the control.

After treatment with indicated concentrations of EFL3 for 48h, the cells were gathered and exposed to double staining and flow cytometry assay. The apoptosis rate was 4.5 ± 3.0 %, 22.0 ± 4.1 %, 35.9 ± 3.2 % for control, 45.0 and 90.0 μM EFL3, respectively (Figure 3). This suggested that EFL3 could induce apoptosis in A549 cells.

**Figure 4:**
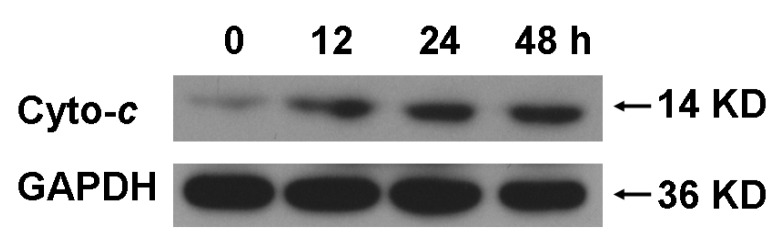
EFL3 treatment induced release of cytochrome *c* (Cyto-*c*) by time-dependent manner after exposure to 90.0 μM EFL3. After A549 cells were exposed to 90.0 μM EFL3 for indicated time, cytosolic part was separated and exposed to western blot to detect cytochrome *c*. GAPDH detection was used to confirm equal protein loading.

Apoptosis is a strictly regulated and organized death process controlling the development and homeostasis of multicellular organisms. Also, apoptosis is related to diseases and drugs-induced pharmacological effects [7]. The mitochondrial pathway plays the important role in apoptosis caused by chemotherapy agents, among which, release of cytochrome *c* from mitochondria into the cytosol is the limiting factor [8,9,10]. Actually, many anticancer drugs can induce apoptosis in susceptible tumor cells via the mitochondrial pathway [6,11]. Indeed, the release of cytochrome *c* was observed after A549 cells were treated with 90.0 μM EFL3 showing time-corresponding pattern (Figure 4). 

**Figure 5:**
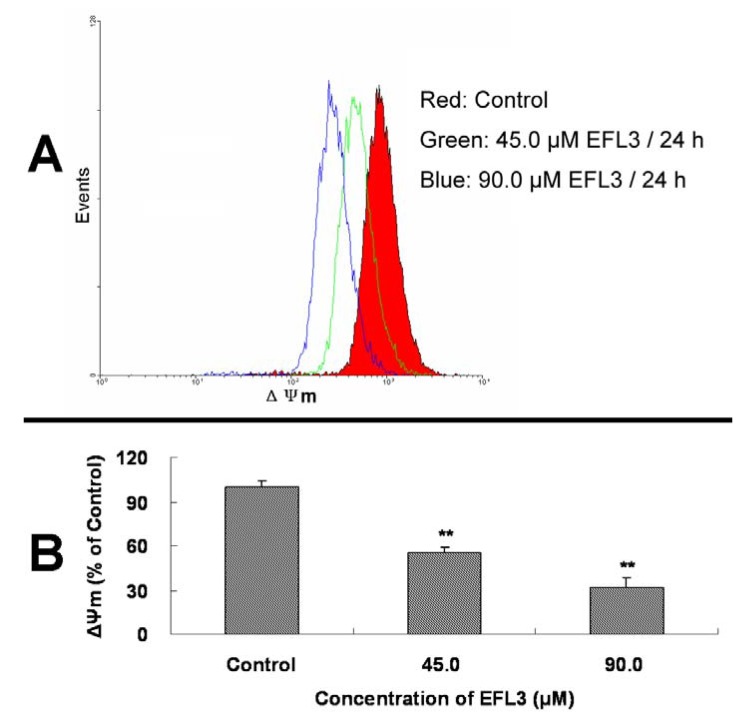
EFL3 treatment caused loss of ΔΨ_m _in A549 cells. **(A)** The decreases of ΔΨ_m_ in A549 cells. **(B)** ΔΨ_m_ levels were calculated as percentage of control. Data were expressed as means ± SD of at least triplicate determinations. ^*^P < 0.05 and ^**^P < 0.01 *vs.* the control.

Mitochondrial dysfunction including loss of ΔΨ_m_ is believed to cause the release of cytochrome *c* [12,13]. To obtain more detail about the apoptosis induced by EFL3, the ΔΨ_m_ of A549 cells before and after treatment of EFL3 was investigated. It is noteworthy that after exposure to 45.0 and 90.0 μM EFL3 for 24 h, the decrease of ΔΨ_m_ compared to the control group was observed showing concentration-dependent manner (Figure 5). Collectively, EFL3 induced loss of ΔΨ_m_ and subsequent release of cytochrome *c* which initiated the apoptosis process.

## 3. Experimental

### 3.1. General

NMR data were recorded on a Varian Inova-400 NB spectrometer (CDCl_3_ as solvent and TMS as internal standard). Chemical shifts (δ) are expressed in ppm with reference to the TMS peak. Mass spectra were acquired on a VG-ZAB mass spectrometer. IR spectra were obtained on a Nicolet 5DX-FTIR spectrophotometer. Column chromatography was performed either on silica gel (200–300 mesh, Qingdao Marine Chemical, Qingdao, PR China) or silica gel H (10–40 μm, Qingdao Marine Chemical, Qingdao, PR China). Fractions were monitored by TLC, visualized by heating silica gel plates sprayed with 5% H_2_SO_4_ in EtOH. Cell viability was measured by Model 550 Microplate reader (BIO-RAD, USA).

### 3.2. Chemicals and reagents

3-(4,5-Dimethyl-2-thiazolyl)2,5-diphenyl-2H-tetrazolium bromide (MTT) and 3,3’-dihexyloxacarbo-cyanine iodide (DiOC6) were purchased from Sigma Chemical Co. Antibodies against GAPDH, anti-mouse IgG-HRP and anti-rabbit IgG-HRP were purchased from KangChen Biotechnology Co. (Shanghai, China). Antibody against cytochrome *c* was purchased from Cell Signalling Biotechnology Co (USA). All tissue culture supplies were purchased from Invitrogen Co. Sephadex LH-20 was purchased from Pharmacia Biotech. Other routine laboratory reagents were obtained from commercial sources of analytical or HPLC grade.

### 3.3. Plants, extraction and isolation

Caper Euphorbia Seed was purchased from Anguo, Hebei province and identified by professor Hu-biao Chen (School of Chinese Medicine, Hong Kong Baptist University). The voucher specimen is deposited at the Herbarium of the Department of Pharmaceutical Sciences, School of Basic Science, Guangzhou Medical College. Powder of Caper Euphorbia Seed (14 kg) was refluxed with 95 % EtOH (4L/kg, 3 h/per time, three times) to obtain the ethanolic extract. Subsequently, the extract was concentrated and suspended in H_2_O and partitioned successively with EtOAc (4 L/per time, three times) and n-BuOH (4 L/per time, three times) to afford corresponding extracts. The EtOAc extract was separated and repeatedly purified by silical gel (Petroleum ether-ethyl acetate) and Sephadex LH-20 column chromatography (methylene chloride-methanol) to afford Euphorbia factor L3 (2.1 g).

### 3.4. Cell lines and cell culture

Human lung cancer A549 cells and human colon cancer LoVo cells were cultured in RPMI 1640 medium containing 100 U/mL penicillin, 100 μM streptomycin and 10% fetal bovine serum (FBS). Human breast cancer MCF-7 cells were cultured in DMEM containing 100 U/mL penicillin, 100 μM streptomycin and 10% fetal bovine serum (FBS). Cells were maintained in a humidified atmosphere incubator containing 5% CO_2_ and 95% air at 37 °C [14]. All experiments were performed during logarithmic growth phase.

### 3.5. Cell viability assay

Cells were harvested during logarithmic growth phase and seeded in 96-well plates at a density of 1.5 × 10^4^ cells/mL of final volume 190 μL/well. After incubation of 24 h, 10 μL EFL3 solution of full range concentrations was added to 96-well plates. After 68 h treatment, 10 μL MTT of 10 mM was added to each well for 4 h of maintaining at 37 °C. Then, the supernatant was removed and the crystals were dissolved with 100 μL anhydrous DMSO each well. Subsequently, cell viability was measured by Model 550 Microplate reader at 540 nm and 655 nm as reference filter. Experiments were carried out at least thrice. The 50% inhibitory concentration (IC_50_) was defined as the anticancer agent concentration causing 50% reduction in cell viability and calculated from the cytotoxicity curves (Bliss’s software). Cell survival was calculated with the following formula: survival (%) = (mean experimental absorbance/mean control absorbance) × 100% [15].

### 3.6. Annexin V-FITC/PI assay of apoptosis

After A549 cells were exposed to the indicated concentrations of EFL3 for 48 h, the cells were gathered and washed twice with ice-cold PBS. Subsequently, annexin V-FITC/PI assay for apoptosis was carried out according to the manufacturer's instruction. The samples were determined by flow cytometer (Becton Dickinson, USA) and analyzed by the CellQuest software. At least 10,000 cells were analyzed for each sample. The apoptosis rate (%) = (the number of apoptotic cells / the number of total cells observed) × 100 % [16]. 

### 3.7. Determination of mitochondrial potential (ΔΨ_m_)

ΔΨ_m_ was determined by flow cytometry and mitochondrial tracking fluorescent DiOC6. After exposure to indicated concentrations of EFL3 for 24 h, 5 × 10^5^ cells were collected, centrifuged at 1,000 rpm for 5 min, and washed with ice-cold PBS once. Thereafter cells were incubated with 40 nM DiOC6 at 37 °C for 20 min in the dark. Subsequently, cells were washed twice with ice-cold PBS, resuspended in 1 mL PBS, and measured by FACS Caliber flow cytometer (Beckman-coulter, Elite) with the excitation wavelength of 484 nm and emission wavelength of 501 nm. At least 10,000 cells were analyzed for each sample. The data obtained from flow cytometry were determined with CellQuest software and expressed as mean fluorescence intensity (MFI). The expressed results were values of at least three independent determinations [17].

### 3.8. Subcellular fractionation for western blot analysis of cytosolic cytochrome c

A549 cells were treated with 90.0 μM EFL3 for indicated time and harvested by centrifugation at 1,000 rpm for 5 min. Then the subcellular fractionation of cytosolic cytochrome *c* was isolated according to the methods reported before. Thus, the protein solution was applied to identification of cytosolic cytochrome *c* by immunoblotting with 12% SDS-PAGE and blotting onto PVDF membrane. The cytochrome *c* protein was detected by anti-cytochrome *c* antibody in the ratio of 1:1,000 [15]. 

### 3.9. Statistical analysis

Results were performed by t-test or one-way ANOVA with SPSS 13.0 software (SPSS Inc., USA). Data were presented as means ± SD of at least triplicate determinations. ^*^P < 0.05 was indicative of significant difference, and ^**^P < 0.01 was indicative of very significant difference.

## 4. Conclusions

Taken together, EFL3 of lathyrane diterpenoids showed potent cytotoxicity to A549 cells. Furthermore, EFL3 induced apoptosis of A549 cells through mitochondrial pathway with involvement of loss of ΔΨ_m_, release of cytochrome *c*.

## References

[1] Shi Q.W., Su X.H., Kiyota H. (2008). Chemical and pharmacological research of the plants in genus Euphorbia. Chem. Rev..

[2] Zhang J.Y., Zhang C., Chen H.B., Fu L.W., Tao Y.W., Zheng X.Q., Cao Z.M., Zhong Y.F., Yu L.H. (2010). Assignments of ^1^H and ^13^C NMR Signals of Euphorbia factor L1 and investigation of itsanticancer activity *in vitro*. J. Med. Plant Res..

[3] Appendino G., Tron G.C., Cravotto G., Palmisano G., Jakupovic J. (1999). An expeditious procedure for the isolation of ingenol from the seeds of euphorbia lathyris. J. Nat. Prod..

[4] Zhang J.Y., Mi Y.J., Chen S.P., Wang F., Liang Y.J., Zheng L.S., Shi C.J., Tao L.Y., Chen H.B., Fu L.W. (2011). Euphorbia factor L1 reverses ABCB1-mediated multidrug resistance involving interaction with ABCB1 independent of ABCB1 downregualtion. J. Cell Biochem..

[5] Gao S., Liu H.Y., Wang Y.H, He H.P., Wang J.S., Di Y.T., Li C.S., Fang X., Hao X.J.  (2007). Lathyranone A: a diterpenoid possessing an unprecedented skeleton from Euphorbia lathyris. Org. Lett..

[6] Rabbani A., Finn R.M., Ausió J. (2005). The anthracycline antibiotics: antitumor drugs that alter chromatin structure. Bioessays.

[7] Strasser A., O'Connor L., Dixit V.M. (2000). Apoptosis signaling. Annu. Rev. Biochem..

[8] Green D.R., Kroemer G. (2004). The pathophysiology of mitochondrial cell death. Science.

[9] Green D.R., Reed J.C. (1998). Mitochondria and apoptosis. Science.

[10] Brenner C., Kroemer G. (2000). Apoptosis. Mitochondria--the death signal integrators. Science.

[11] Zhang J.Y., Tao L.Y., Liang Y.J., Chen L.M., Mi Y.J., Zheng L.S., Wang F., She Z.G., Lin Y.C., To K.K., Fu L.W. (2010). Anthracenedione derivatives as anticancer agents isolated from secondary metabolites of the mangrove endophytic fungi. Mar. Drugs.

[12] Viola G., Fortunato E., Cecconet L., Disarò S., Basso G. (2007). Induction of apoptosis in Jurkat cells by photoexcited psoralen derivatives: Implication of mitochondrial dysfunctions and caspases activation. Toxicol. In Vitro..

[13] Susnow N., Zeng L., Margineantu D., Hockenbery D.M. (2009). Bcl-2 family proteins as regulators of oxidative stress. Semin Cancer Biol..

[14] Zhang J.Y., Tao L.Y., Liang Y.J., Yan Y.Y., Dai C.L., Xia X.K., She Z.G., Lin Y.C., Fu L.W. (2009). Secalonic acid D induced leukemia cell apoptosis and cell cycle arrest of G(1) with involvement of GSK-3beta/beta-catenin/c-Myc pathway. Cell Cycle..

[15] Zhang J.Y., Wu H.Y., Xia X.K., Liang Y.J., Yan Y.Y., She Z.G., Lin Y.C., Fu L.W. (2007). Anthracenedione derivative 1403P-3 induces apoptosis in KB and KBv200 cells via reactive oxygen species-independent mitochondrial pathway and death receptor pathway. Cancer Biol. Ther..

[16] Yan Y.Y., Su X.D., Liang Y.J., Zhang J.Y., Shi C.J., Lu Y., Gu L.Q., Fu L.W. (2008). Emodin azide methyl anthraquinone derivative triggers mitochondrial-dependent cell apoptosis involving in caspase-8-mediated Bid cleavage. Mol. Cancer Ther..

[17] Wang X.H., Jia D.Z., Liang Y.J., Yan S.L., Ding Y., Chen L.M., Shi Z., Zeng M.S., Liu G.F., Fu L.W. (2007). Lgf-YL-9 induces apoptosis in human epidermoid carcinoma KB cells and multidrug resistant KBv200 cells via reactive oxygen species-independent mitochondrial pathway. Cancer Lett..

